# The Use of MRI to Detect Malignancy in a Patient Presenting With Unilateral Bloody Nipple Discharge

**DOI:** 10.7759/cureus.47986

**Published:** 2023-10-30

**Authors:** Kaitlyn N Romero, Taylor Ouellette, Radhika Patel, Trishna Patel

**Affiliations:** 1 Internal Medicine, Nova Southeastern University Dr. Kiran C. Patel College of Osteopathic Medicine, Fort Lauderdale, USA; 2 General Surgery, Nova Southeastern University Dr. Kiran C. Patel College of Osteopathic Medicine, Fort Lauderdale, USA; 3 Breast Imaging, Baptist Health, Jacksonville, USA

**Keywords:** pathological nipple discharge, mri breast, nipple discharge, breast cancer detection, diagnostic mammography

## Abstract

Nipple discharge presents as either physiological, which is green, white, or yellow, or is considered pathological, which is typically unilateral, spontaneous, and bloody. Bloody nipple discharge (BND) can be due to underlying malignancy or premalignant lesions. Mammogram (MMG), ultrasound (US), MRI, and ductography are all used to evaluate BND, but different modalities offer greater value in the diagnostic process. Here, we present a case that demonstrates the ability of MRI to detect abnormalities not seen on MMG and US in the setting of BND due to underlying malignancy. The use of MRI earlier in the diagnostic process allows for the use of breast-conserving measures and decreases the possibility of metastasis. This would result in less of a need for more aggressive treatments.

## Introduction

Nipple discharge presents in approximately 3-10% of women and is classified as either physiological or pathological. Physiologic discharge can be green, white, or yellow and presents bilaterally and non-spontaneously [1]. Benign papilloma is the most common etiology of pathologic nipple discharge [2]. Unilateral, spontaneous bloody nipple discharge (BND) presents due to an underlying malignancy in 5-21% of cases with ductal carcinoma in situ (DCIS) being the most common malignancy [2]. BND can be the initial presentation for papillary lesions, ductal ectasia, periductal mastitis, nipple adenoma, Paget’s disease, DCIS, and even invasive ductal or lobular carcinoma [1].

The American College of Radiology (ACR) does not recommend imaging for physiological discharge but recommends a mammogram (MMG) and ultrasound (US) as first-line imaging modalities for BND. For women less than 30 years of age who present with BND, they should first undergo US. If family history is present or if the US revealed a suspicious finding, then proceeding to an MMG is recommended. For women between 30 and 39 years of age with BND, beginning first with MMG is recommended and, if necessary, then US. Women greater than 40 years of age with pathologic nipple discharge are recommended to have both an MMG and US [2].

Ductography and MRI are used to further rule out benign, premalignant lesions, such as intraductal papillomas [3]. The detection of breast cancer is based on angiogenesis, and after one to two minutes of administration of gadolinium, there is prompt uptake and release due to the increased permeability of tumor blood vessels [4]. Studies show MRI has a 93-100% sensitivity for detecting invasive breast cancer but is not generally used unless suspicion for malignancy remains high after MMG and US are inconclusive [1-4]. Ductography is an invasive procedure that can cause discomfort and pain and can only be used when discharge is present as the duct that is draining is cannulated and filled with dye to allow for evaluation. It is more sensitive than MMG and US; however, it has lower specificity [1,5-6].

Currently, MRI in breast imaging has specific indications. Women with genetic-based increased risk, Tyrer-Cuzick score greater than 20%, history of chest or mantle radiation, history of breast cancer, and heterogeneously or dense breasts are recommended to have a contrast-enhanced MRI [2,7]. Compensation by insurance can be a barrier to obtaining MRIs for patients outside of these indications [7].

This case report presents a patient with non-invasive breast malignancy that was undetectable on MMG and benign-appearing on US but fully appreciated with the use of MRI. The case report was presented in the American Medical Association Research Challenge on October 22, 2022.

## Case presentation

A 53-year-old female with no significant past medical history presented for diagnostic MMG after complaints of focal right breast pain and BND on expression. She described the pain as an episode of obstructed milk ducts. The patient is gravida 2 para 2, with her first pregnancy before the age of 30 years old and menarche at age 11. The patient breastfed following both of her pregnancies. She has a history of 15 years of oral contraceptive use and no use of hormonal replacement therapy.

The patient has a history of benign-appearing calcifications in the right upper outer quadrant seen in 2019 on digital bilateral tomosynthesis (DBT). DBT was done in 2019 following a complaint of left-sided focal breast pain. There were no abnormal findings in the left breast and no other findings in the right breast besides the benign-appearing calcifications. Family history includes a paternal aunt diagnosed at the age of 63 with HER-2+ invasive ductal carcinoma. The Tyrer-Cuzick score of 22.6% was calculated for the patient and her breast density was categorized as a BI-RADS-C; therefore, MRI of the breast was recommended for heterogeneously dense breasts in 2019, which was not completed as the patient did not follow up.

**Figure 1:**
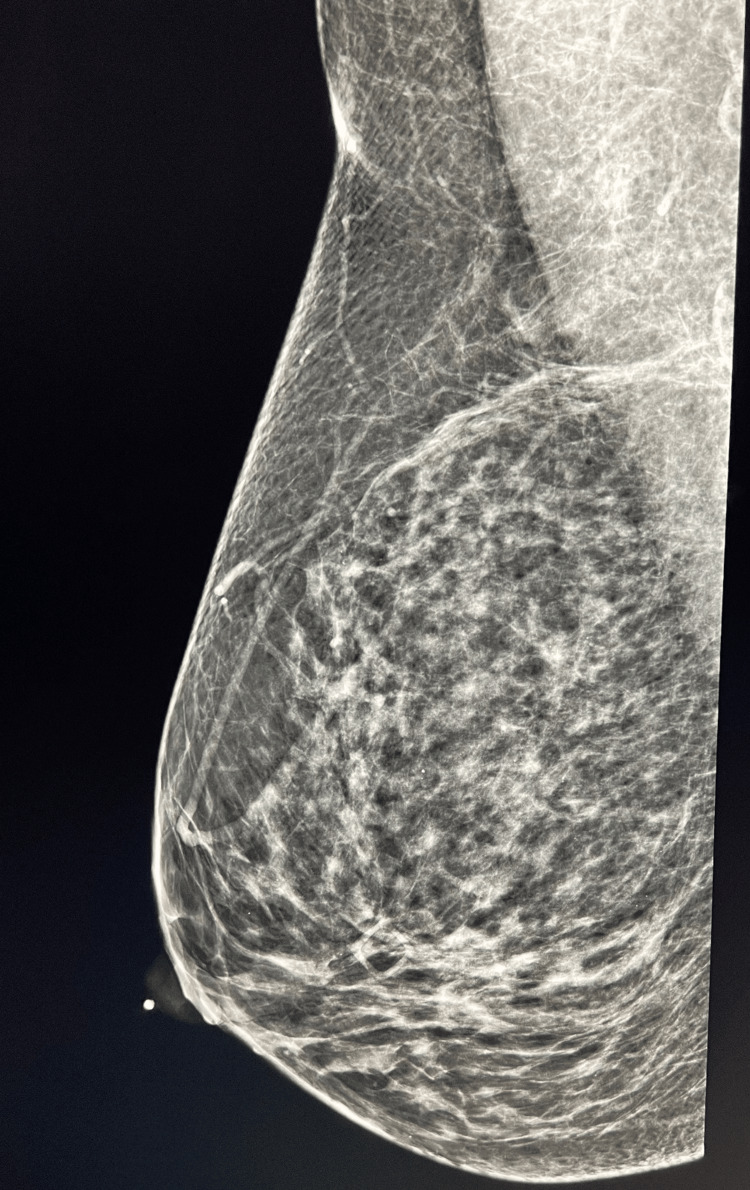
Mediolateral oblique of the right breast on DBT Square box: area of focal pain on the lateral breast

**Figure 2:**
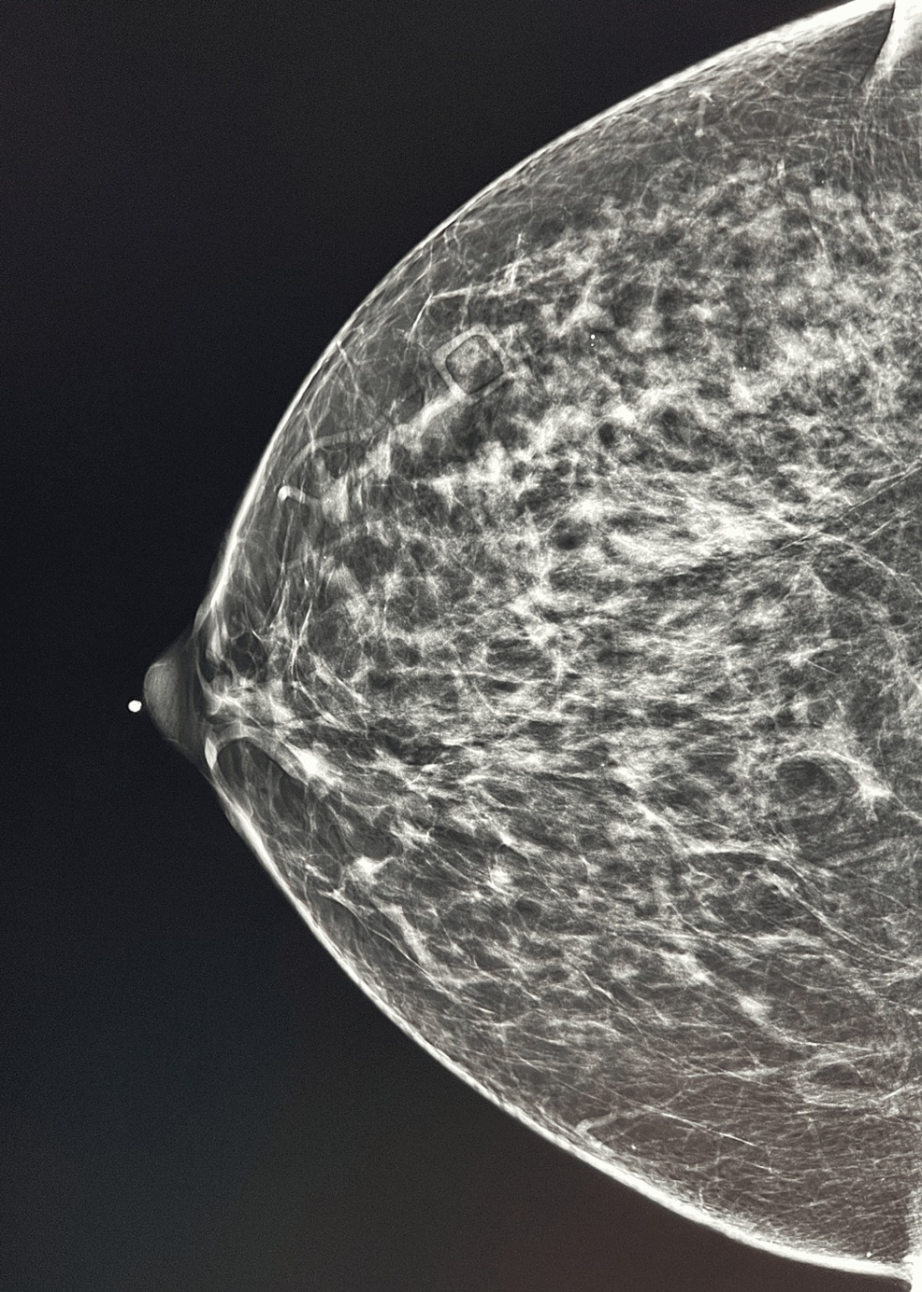
Craniocaudal view of the right breast on DBT Square box: area of focal pain on the lateral breast

In 2021, DBT was completed after an episode of right, unilateral bloody discharge that was non-spontaneous and pain that she compared to an episode of obstructed milk ducts. DBT showed the same stable calcifications in the right upper outer quadrant from 2019 and no abnormalities in the region of the BND or focal pain in the right breast, as seen in Figures 1-2. A BI-RADS category 4A indicated a low suspicion for malignancy, and unilateral diagnostic follow-up MMG of the right breast was recommended.

**Figure 3:**
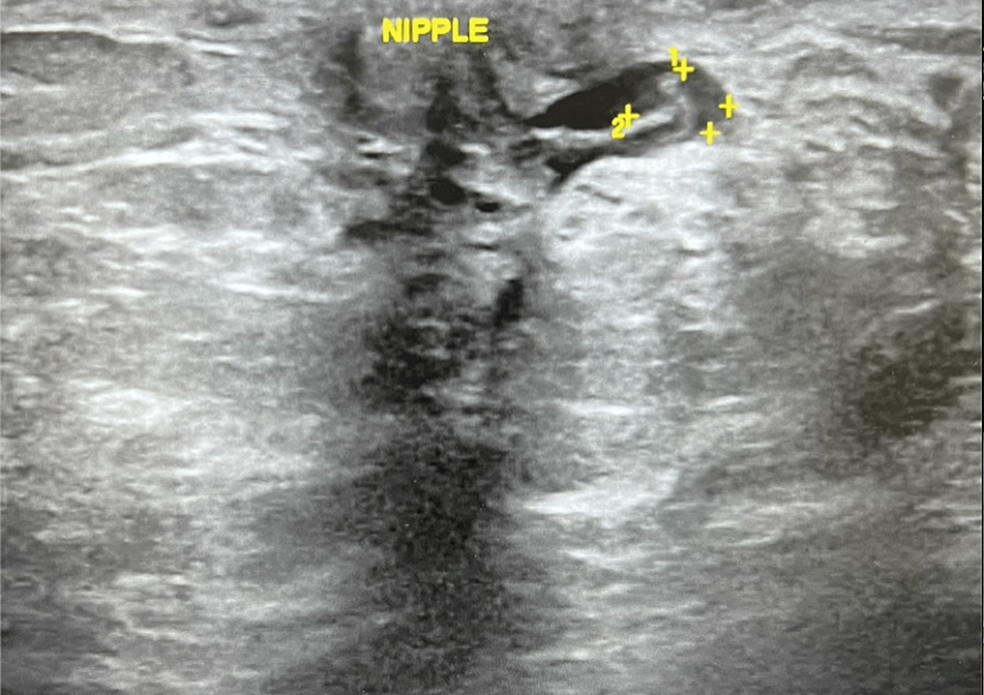
US demonstrating 5x3x3 mm subareolar mass at the 6 o’clock position in the right breast

**Figure 4:**
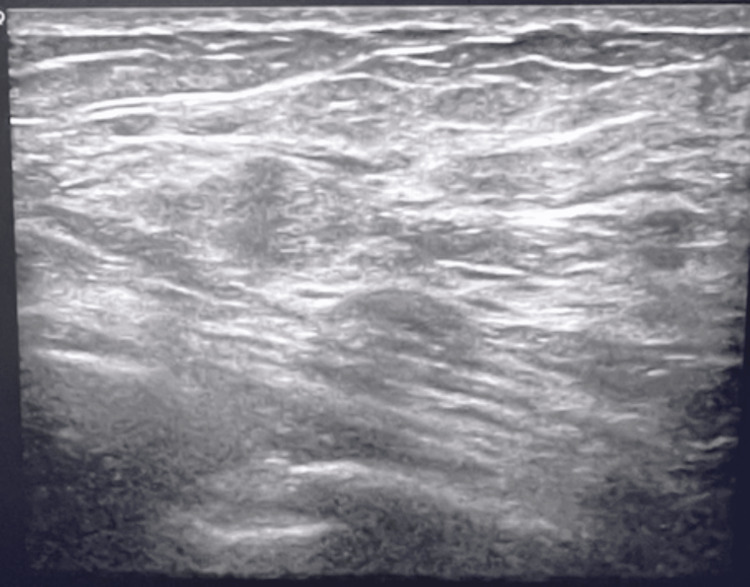
Negative US of the right breast area of focal pain at 8 o'clock, 6 cm from the nipple

Following DBT, US was performed on the right breast at the 6 o'clock position, demonstrating a 5.0x3.0x3.0 mm subareolar irregular mass correlating to BND (Figure 3). There was no sonographic finding correlating with the area of focal pain at 8 o'clock, 6 cm from the nipple shown in Figure 4. US-guided biopsy of the subareolar mass was recommended and performed prior to MRI. The pathology results of the biopsy were significant for atypical ductal hyperplasia (ADH), intraductal papilloma, columnar cell changes, and apocrine metaplasia.

**Figure 5:**
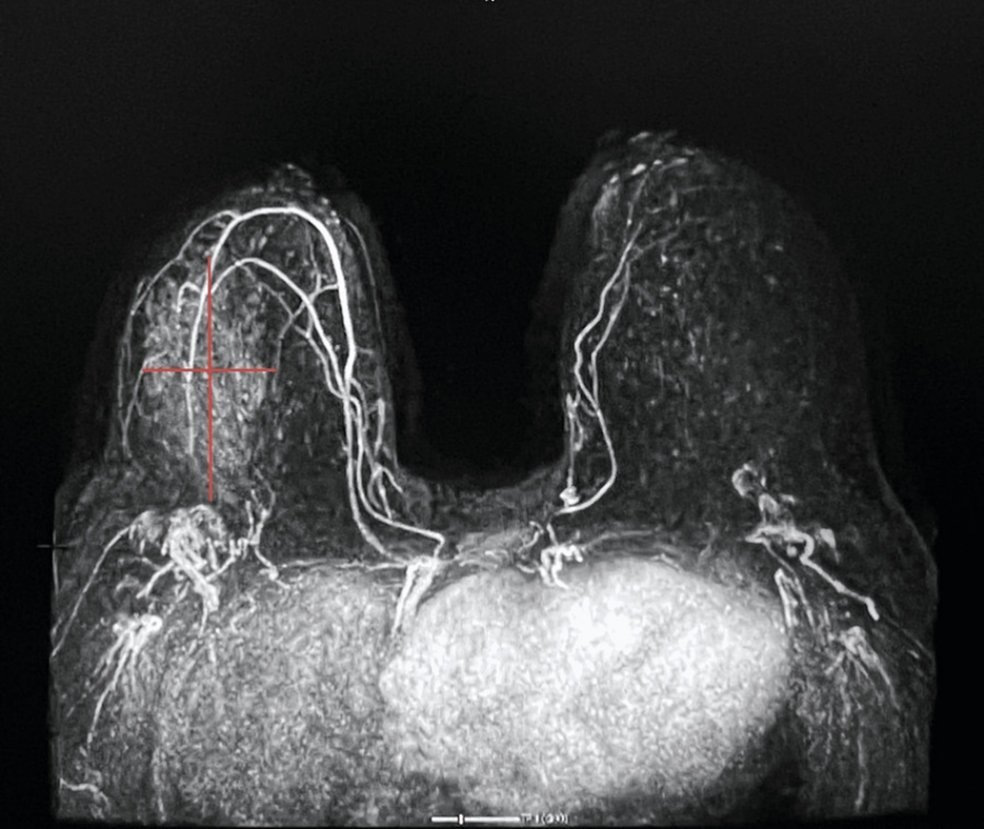
Axial MRI of the breast showing a 9x10.5x4.6 cm non-mass-like enhancement in the right breast

**Figure 6:**
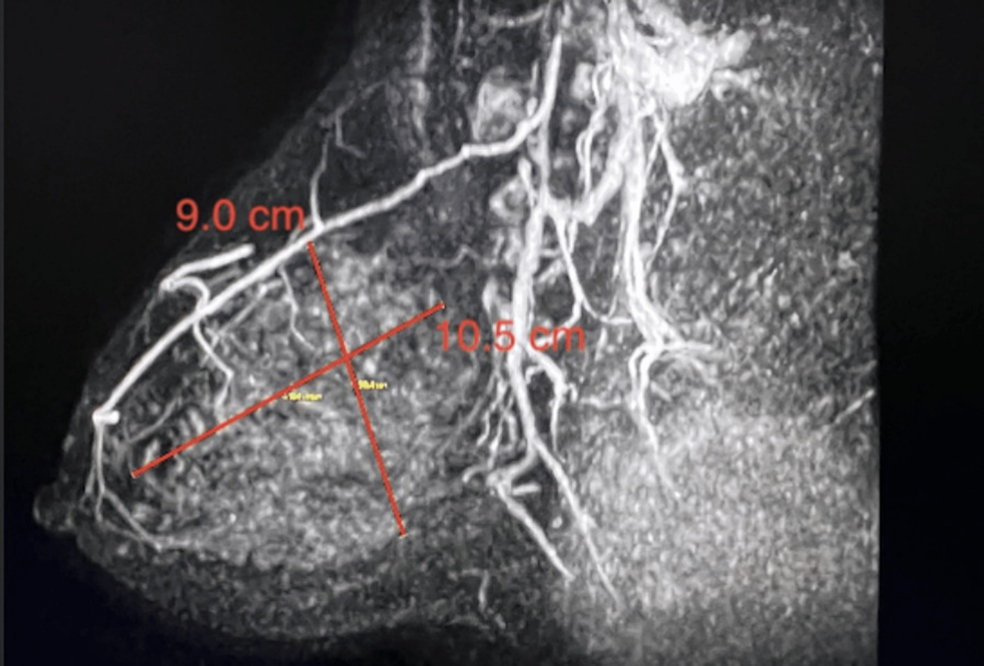
Lateral MRI of the breast showing a 9x10.5x4.6 cm non-mass-like enhancement in the right breast

Following DBT and US, a high-risk MRI one month later demonstrated non-mass-like enhancement extending 2-11 cm from the nipple in the right breast. The non-mass enhancement extended approximately 10.5x4.6x9.0 cm. Faint microcalcifications were diffusely distributed in the upper outer and lower outer quadrants of the right breast and served as targets for tissue diagnosis. Figures 5-6 demonstrate the non-mass enhancement shown on MRI.

After diagnostic imaging and MRI revealing the extent of abnormality in the right breast, a stereotactic biopsy of the upper outer quadrant was conducted. Stereotactic biopsy was used because the calcifications were first visualized on DBT in 2019 and then in 2021, correlating with the faint microcalcifications found on MRI. The pathology findings included DCIS, ADH, and intraductal papilloma, with estrogen receptor and progesterone receptor positivity. The findings were positive for malignancy, but no invasive component was seen by pathology. Simultaneously, the patient underwent a stereotactic core biopsy of calcifications in the lower outer quadrant, demonstrating benign pathology results.

The patient underwent a total mastectomy of the right breast and was not a candidate for breast-conserving therapy due to the size of the mass seen on MRI and the malignant findings confirmed by biopsy. The procedure took place two months following the initial presentation of BND. Surgical pathology confirmed DCIS with microinvasion and tumor staged T1N0M0. The specimen comprised approximately 35% of the lateral breast, with positive margins. Since undergoing unilateral mastectomy and systemic chemotherapy over 14 months ago, the patient has had no recurrence of cancer.

## Discussion

Currently, indications for MRI as a screening tool are considered in high-risk patients. In patients with BND, MRI is used when initial imaging is inconclusive on MMG and US, but clinical suspicion remains high [7,8]. MRI not only demonstrates earlier detection of malignant and premalignant lesions but can detect lesions that are not visualized or fully appreciated with MMG and US. The patient in the case report was initially presented with benign-appearing microcalcifications categorized as a BI-RADS-3 and was recommended to follow up with diagnostic MMG for six months. MRI was recommended based on her Tyrer-Cuzick score of 22.6%. MRI, although not recommended for the macrocalcifications seen on DBT, uncovered the full extent of the disease despite inconclusive US and MMG with benign-appearing findings. Ductography was not regularly performed at this center.

DCIS presents in 90% of women as suspicious microcalcifications found on MMG. Patterns of microcalcifications seen on MMG that suggest high-nuclear-grade DCIS include segmental types of pleomorphic microcalcifications and linear branching, while cribriform pattern, low-grade, or micropapillary DCIS is associated with more fine, granular calcifications [4]. According to a study conducted by Lehman et al., the sensitivity of MMG was 85.3% and the specificity was 96.4% for malignancy [9]. However, in multifocal disease, the extent of DCIS may be underestimated by MMG [4,9].

MMG and US may not display lesions if they are too small, contain no calcifications, or are completely intraductal, such as in lobular carcinomas [10]. The ACR reports that the sensitivity of MRI in detecting invasive breast cancer is high, ranging from 93% to 100% in multiple studies [2]. In a study of 106 females with BND and MMG and US categorized as BI-RADS-1, an MRI was done, of which 54 of the studies displayed abnormal enhancement. Of the 54, 46 underwent excision of lesions and included eight cancers and five papillomas with atypia. The results of the study showed accuracy for the detection of lesions requiring excision to have a 96% sensitivity and 98% specificity [3]. Saadatmand et al. conducted a large multicenter randomized control trial that found more breast cancers were detected in the group using MRI for screening. Cancers were overall smaller in size, with earlier tumor staging, and less likely to have metastasized to lymph nodes in the group screened with MRI [10].

MRI has multiple advantages to current imaging modalities used in the work-up of BND. Compared to ductograms that involve the cannulation of single ducts, MRI can visualize the entire breast, including the retroareolar region, and is a minimally invasive approach [1]. A study comparing MRI versus ductograms showed MRI superior in detecting lesions with 85% versus 77% in the group undergoing ductograms [6]. Ductograms have fallen out of favor in current practice due to a number of reasons, including the need for discharge to be present and the discomfort it causes to the patient [5,6].

There is limited data comparing MMG and MRI in patients with an initial presentation of BND. Future studies should focus on the use of MRI as first-line imaging in patients presenting with BND due to the greater ability of MRI to detect abnormal breast tissue and display the full extent of malignancy. If MRI is negative, malignancy can likely be ruled out. As MRIs become more affordable, and more importantly, as scan times decrease, hopefully, the increased utilization of MRIs can lead to earlier and improved detection of breast cancer. Until then, recognition of clinical scenarios, such as BND, where MRI has better sensitivity, should be considered in the work-up.

## Conclusions

Diagnostic work-up of BND includes MMG, US, and invasive measures such as ductoscopy. Major duct excision can remove affected lobules but is an invasive procedure. Patients more often undergo lumpectomy or mastectomy. However, this case demonstrates the ability of MRI to fully detect abnormalities not detected on DBT and US. The use of MRI earlier in the diagnostic process allows more breast-conserving measures, such as lumpectomy and radiation therapy versus systemic chemotherapy and mastectomy. Moreover, earlier detection decreases the chances of invasion and the possibility of metastasis. This would result in less need for more aggressive treatments and better visualization of cancers.
